# Family and Population-Based Studies of Variation within the Ghrelin Receptor Locus in Relation to Measures of Obesity

**DOI:** 10.1371/journal.pone.0010084

**Published:** 2010-04-09

**Authors:** Anette P. Gjesing, Lesli H. Larsen, Signe S. Torekov, Irena Aldhoon Hainerová, Rahul Kapur, Anders Johansen, Anders Albrechtsen, Sylvia Boj, Birgitte Holst, Angela Harper, Søren A. Urhammer, Knut Borch-Johnsen, Charlotta Pisinger, Søren M. Echwald, Hans Eiberg, Arne Astrup, Jan Lebl, Jorge Ferrer, Thue W. Schwartz, Torben Hansen, Oluf Pedersen

**Affiliations:** 1 Steno Diabetes Center, Gentofte, Denmark; 2 Hagedorn Research Institute, Gentofte, Denmark; 3 Institute of Human Nutrition, University of Copenhagen, Copenhagen, Denmark; 4 Department of Paediatrics and Centre for Research of Diabetes, Metabolism and Nutrition, 3rd Faculty of Medicine, Charles University, Prague, Czech Republic; 5 Endocrinology Unit, Hospital Clinic de Barcelona, Institute d'Investigacions Biomediques August Pi i Sunyer, Barcelona, Spain; 6 Laboratory for Molecular Pharmacology, Department of Pharmacology, Panum Institute, University of Copenhagen, Copenhagen, Denmark; 7 Institute of Human Nutrition, Royal Veterinary and Agricultural University, Copenhagen, Denmark; 8 Department of Endocrinology, Hvidovre University Hospital, Hvidovre, Denmark; 9 Research Centre for Prevention and Health, Glostrup University Hospital, Glostrup, Denmark; 10 Exiqon, Vedbæk, Denmark; 11 Department of Cellular and Molecular Medicine, University of Copenhagen, Copenhagen, Denmark; 12 Faculty of Health Sciences, University of Copenhagen, Copenhagen, Denmark; 13 Faculty of Health Science, University of Aarhus, Aarhus, Denmark; 14 Faculty of Health Sciences, University of Southern Denmark, Odense, Denmark; 15 Department of Pediatrics, Second Faculty of Medicine, Charles University, Prague, Czech Republic; National Institute of Child Health and Human Development/National Institutes of Health, United States of America

## Abstract

**Background:**

The growth hormone secretagogue receptor (GHSR) is mediating hunger sensation when stimulated by its natural ligand ghrelin. In the present study, we tested the hypothesis that common and rare variation in the *GHSR* locus are related to increased prevalence of obesity and overweight among Whites.

**Methodology/Principal Findings:**

In a population-based study sample of 15,854 unrelated, middle-aged Danes, seven variants were genotyped to capture common variation in an 11 kbp region including *GHSR*. These were investigated for their individual and haplotypic association with obesity. None of these analyses revealed consistent association with measures of obesity. A -151C/T promoter mutation in the *GHSR* was found in two unrelated obese patients. One family presented with complete co-segregation, but the other with incomplete co-segregation. The mutation resulted in an increased transcriptional activity (p<0.02) and introduction of a specific binding for Sp-1-like nuclear extracts relative to the wild type. The -151C/T mutation was genotyped in the 15,854 Danes with a minor allele frequency of 0.01%. No association with obesity in carriers (mean BMI: 27±4 kg/m^2^) *versus* non-carriers (mean BMI: 28±5 kg/m^2^) (p>0.05) could be shown.

**Conclusions/Significance:**

In a population-based study sample of 15,854 Danes no association between *GHSR* genotypes and measures of obesity and overweight was found. Also, analyses of *GHSR* haplotypes lack consistent associations with obesity related traits. A rare functional *GHSR* promoter mutation variant was identified, yet there was no consistent relationship with obesity in neither family- nor population-based studies.

## Introduction

The growth hormone secretagogue receptor (GHSR) is a G-protein coupled, seven-transmembrane receptor. It was an orphan receptor until 1999 when its natural ligand, ghrelin, was identified [1]. Ghrelin is a peptide hormone secreted from gastric cells in response to absence of food in the stomach [1]. The ghrelin/GHSR system has many functions. One of these is the signal transduction of hunger by secretion of ghrelin from an empty stomach, stimulating GHSR and leading to the sensation of hunger [2]. As a consequence, selectively knocking out the GHSR in the arcuate nucleus in rodents results in lower body weight and a decrease in adipose tissue [3]. In addition, a reduction in food intake in mice receiving GHSR antagonists was observed [4]. Stimulation of hunger by the ghrelin/GHSR system is mediated by neurons in the hypothalamic arcuate nucleus; in particular, neurons expressing neuropeptide Y and agouti-related protein [5]. In fact, this effect of the ghrelin/GHSR system on hunger is considered central for appetite regulation, as the GHSR has high constitutive activity, which may be a set-point in appetite regulation, counteracting the anorexigenic hormones leptin and insulin [6].

The gene encoding *GHSR*, which is evolutionary highly conserved, is located on chromosome 3q26, a region previously showing linkage to measures of body composition [7]. A comprehensive analysis of single nucleotide polymorphisms (SNPs) and haplotype structure across the entire *GHSR* region (99.3 kb) identified a linkage disequilibrium (LD) block consisting of five SNPs showing linkage with body mass index (BMI) in an intrafamilial segregation study among 178 obese pedigrees (Whites), as well as association with obesity at the population level among 1,418 Whites [8]. Common variants in *GHSR* were also associated with obesity and obesity-related traits in a French case-control study of 602 subjects; yet replication of such an association in a German study sample of 888 individuals failed [9]. Furthermore, studies of the functional rare mutations, Ala20Glu and Phe279leu, indicated a co-segregation with obesity [10], [11].

In light of these previous findings, we investigated the effect of seven common single nucleotide polymorphisms (SNPs) on obesity at the population level. We also examined the effect of rare variants by screening the promoter and coding regions of the *GHSR,* and identified a rare gain-of-function mutation in the *GHSR* promoter. The biochemical effect of this variant was assessed *in vitro* and the physiological effect was investigated among Danish and Czech individuals with a familial predisposition for obesity and in a population-based study sample.

## Methods

### Study materials–population studies

The population of Danes used in this study were recruited from the following study groups: 1) Inter99: 6,514 individuals from a population-based randomised non-pharmacological intervention study for prevention of cardiovascular disease conducted at the Research Centre for Prevention and Health in Copenhagen County (ClinicalTrials.gov ID-no:NCT00289237 [12]). 2) SDC: 676 unrelated middle-aged individuals from a population based sample recruited at Steno Diabetes Center. 3) Addition: 8,664 individuals from a high-risk screening study for type 2 diabetes recruited from Department of General Practice at the University of Aarhus, Aarhus, Denmark (ClinicalTrials.gov Identifier: NCT00237548 [13]). Cases were defined as having a BMI above 30 kg/m^2^ and controls as having a BMI below 25 kg/m^2^. Of the 4,217 individuals having a BMI above 30 kg/m^2^ 1,136 were from study group 1, 175 were from study group 2 and 2,906 were from study group 3. Of the 3,217 individuals having a BMI below 25 kg/m^2^ 2,831 were recruited from study group 1 and 386 were from study group 2. Individuals from study group 3 with a BMI below 25 kg/m^2^ were excluded from the case-control studies as this study group 3 was collected based on a high risk of having or developing type 2 diabetes, thus even people who were lean are not likely to be representative of the general population of lean control individuals. All study participants were Danes by self-report.

### Mutation screening

82 obese probands with a mean BMI of 35.5± standard deviation (SD) of 4.6 kg/m^2^ were selected from families with a least two overweight individuals recruited at the outpatient clinic at Steno Diabetes Center. 28 (7 men/21 women) lean subjects with a BMI of 20.0±1.7 kg/m^2^ were from study group 2. Mutation identification was performed using single-strand conformation polymorphism of the coding region (including intron-exon boundaries) along with 378 base pairs of the minimal promoter relative to the ATG site of *GHSR* (NCBI accession number: AY322544 and AF369786) on DNA from Danish probands. Primers and conditions are available by request to corresponding author.

Screening for the -151C/T *GHSR* variant was also performed in 289 (157 girls/132 boys) unrelated obese Czech children, aged 1–18 years by PCR-RFLP. Inclusion criteria were obesity-onset before the age of 11 years and BMI above the 97^th^ percentile for sex and age according to Czech national references [14]. The average age of obesity-onset was 4.9±3.1 years and the average Z-score for BMI at the time of recruitment was 4.3±1.7. *MC4R* mutations in the -151C/T *GHSR* variant carriers as a cause of obesity were excluded by sequencing.

### Family studies

Family members of the Danish and of the Czech probands carrying the *GHSR* -151 C/T promoter mutation were examined for the presence of this variant using sequencing.

### Ethics statement

Informed written consent was obtained from all participants (or legal guardian if under 18 years) and the study protocols were approved by the Ethics Committee of the 3rd Faculty of Medicine, Charles University in Prague or the regional Ethics Committees in Denmark (ethics committee, Copenhagen County for the Inter99 and the SDC and ethics committee, Aarhus County for the Addition) and the study was conducted in accordance with the Helsinki declaration II.

### Anthropometrics, behavior and biochemical assays

#### Population studies

Height and weight were measured in light indoor clothes and without shoes, and BMI was calculated as weight (kg)/(height (m))^2^. Blood samples for biochemical analyses were drawn in the morning after an overnight fast. Plasma glucose and serum specific insulin (and intact proinsulin) were analysed using Steno Diabetes Center standard methods [12]. Serum total and HDL cholesterol were analysed using enzymatic colorimetric methods (GPO-PAP and CHOD-PAP, Roche Molecular Biochemicals, Germany).

#### Family studies

Height and weight were measured as described above. After a minimum 12 hour fast, carriers of the -151C/T mutation were examined for circulating levels of fasting glucose, triglycerides, total cholesterol, free fatty acids, leptin, luteinizing hormone (LH), follicle stimulating hormone (FSH), growth hormone, IGF-1 and IGF-BP3 by standard methods at the Institute for Human Nutrition, Copenhagen, Denmark or the Department of Paediatrics and Centre for Research of Diabetes, Metabolism and Nutrition, 3rd Faculty of Medicine, Charles University, Prague, Czech Republic. The habitual eating behavior of the subjects was assessed by use of a three-factor eating questionnaire (TFEQ), which measured dietary restraint, disinhibition and hunger [15]. A meal test was conducted in three Danish family members, DK-III-2, DK-II-2 and DK-IV-1. The breakfast was given after an overnight fast and spontaneous energy intake during an *ad libitum* lunch meal was measured 4.5 hours later. The breakfast had a fixed size and energy content, consisting of yogurt, bread, butter, cheese, jam, kiwi-fruit, orange juice and water with a total energy content equivalent to 20% of each subject's 24 hours energy requirement [16]. The lunch consisted of pasta, minced beef, sweet corn, carrots, green peppers, onions, courgettes and cream. The distribution of energy in both meals was 50 energy-percent (E%) carbohydrates, 37 E% fat and 13 E% protein. Subjects had followed a weight-maintaining standardised diet containing the same energy distribution two days prior to the test day. The energy and nutrient composition of the test meals and diets were calculated using the DANKOST 2 program, based on the Danish food composition tables [17]. Food items were prepared at the Department of Human Nutrition.

### Genotyping

Common SNPs needed to capture the locus variation in a region including 2 kb upstream and 5.5 kb downstream of *GHSR* (approx. 11 kb) were selected using pair wise tagging of SNPs with a minor allele frequency (MAF) >0.05 with R^2^>0.8. The SNPs selected were: rs1403637, rs1916345, rs2948694, rs572169, rs2922126, rs495225, rs509035. These SNPs and the -151C/T promoter variant were genotyped using chip-based matrix-assisted laser desorption/ionisation time-of-flight mass spectrometry (DNA Mass-ARRAY; Sequenom, San Diego, CA) of PCR-generated primer extension products as described earlier [18] or using Taqman allelic discrimination (KBioscience, Hoddesdon, UK). All genotypes were in Hardy-Weinberg equilibrium (p>0.08). The genotypic success of *GHSR* tag SNPs was above 95.5% with a mismatch frequency below 0.3%. The *GHSR* -151C/T variant had a genotype success rate of 92.6% and an error rate of 0.2%.

### *In vitro* analysis of the *GHRS* -151C>T mutation

Two constructs with the promoter segment spanning from −643 to −1 bp with and without the mutation (referred to as 643-Mutant and 643-Wild type) were prepared by amplification of DNA from one carrier and one non-carrier of the -151C>T mutation. Subsequently, introduction of *Bgl*II and *Hind*III sites (New England Biolabs) and site-directed mutagenesis was followed by digestion and ligation of those sites to a pGL3 Basic Vector (Promega). After ligation all constructs were bidirectionally sequenced (MWG-biotech). Primers, restriction-enzyme-cleavage-site-generating-primers and PCR conditions are available on request from the corresponding author.

Rat mammosomatotrophic pituitary tumor cell lines, GH1 (ATCC) and GH4 (ATCC), were maintained in DMEM 1885 with 10% FBS, 1% glutamine and 1% penicillin-streptomycin. The ghrelin-receptor-promoter-pGL3 constructs, pGL3-Basic Vector (Promega) and pGL3-Promoter Vector (Promega) were transfected into the GH1 and GH4 cells. The constructs were co-transfected with CMV-Renilla Control Vector (Promega). Cells were transfected with 50 ng luciferase construct and 50 ng renilla construct in wells with 50,000 (GH1) and 40,000 (GH4) cells, respectively. The luciferase/renilla assay was performed using Firelite dual luminescence reporter gene assay system Kit (PerkinElmer) according to manufacture's protocol. Luciferase activity of each construct was normalised with the correspondent renilla activity, and values were expressed relative to the activity of the basic construct. The experiments were repeated 4 times and each experiment represents the mean of 6 replicates. Electro mobility shift assays (EMSAs) were performed with nuclear extracts from GH4 cells incubated with labeled oligonucleotide probes containing a Sp1 consensus site, the wild type *GHSR* −151C or the mutated *GHSR* −151T sequence. Anti-Sp1 antibody was added to the reaction mix. The binding to the −151T probe was competed with 200-fold molar excess of unlabeled probe.

### Statistical analyses

Fisher's exact test and logistic regression were applied to test for significant differences in allele frequencies and genotype distribution in the obesity case-control studies adjusted for age and sex. A multiple linear model was used to test for difference between genotype groups assuming an additive model in quantitative trait studies. The genotype-quantitative trait study was performed after excluding patients with known type 2 diabetes (Jørgensen et al, 2003). In the present study population a high LD (R^2^ = 0.96) between rs509035 and rs572169 was observed and data for rs509035 are therefore not shown (Figure S1).

An expectation-maximisation (EM) algorithm was applied to estimate the haplotype frequencies used in the association studies. The global p-value is an estimate of the overall effect of haplotypes in the statistical model and the specific p-value is an estimate of the effect of a specific haplotype compared to the effect of the remaining haplotypes combined [19]. Haplotypes with a frequency below 5% were excluded.

Analyses were performed using Statistical Package for Social Science (SPSS, Chicago, Ill., USA) version 12.0 and RGui version 2.7.0 except for analyses of the activity difference between mutant and wild type constructs which was calculated using a t-test for paired samples. A *p*-value of less than 0.05 was considered significant.

## Results

### HapMap-based population studies of common *GHSR* variants

To clarify if common variations in *GHSR* associate with obesity in a Danish study population we genotyped seven SNPs in *GHSR* including a region of 11 kbp (HapMap build 35; region: 173640000–173651000 bp). None of the SNPs were associated with obesity (Table S1) or related quantitative traits (BMI, weight, waist circumference and waist-to-hip ratio) (Table 1).

**Table 1:** Studies of associations between *GHSR* -151 promoter variant and quantitative traits in 15,854 Danes without known type 2 diabetes.

	Wild type	Heterozygous	Homozygous	P_ADD_
-151 promoter variant				
**N (M/W)**	14271(7473/6798)	26(17/9)	-	-
**Age (years)**	54±10	55±9	-	-
**BMI (kg/m^2^)**	27.5±4.9	26.5±3.8	-	0.2*
**Weight (kg)**	81.1±16.3	79.1±14.6	-	0.2*
**Waist (cm)**	92.6±14.3	92.3±12.0	-	0.4*
**Waist-to-hip ratio**	0.86±0.09	0.88±0.08	-	0.6*
**Rs1403637**				
**N (M/W)**	5768(3039/2729)	6947(3645/3302)	2190(1149/1041)	
**Age (years)**	54.5±9.9	54.5±10.0	54.4±9.9	
**BMI (kg/m^2^)**	27.6±4.8	27.6±4.9	27.5±4.9	0.7
**Weight (kg)**	81.1±16.2	81.0±16.3	81.0±16.3	0.8
**Waist (cm)**	92.6±14.3	92.6±14.4	92.4±14.1	0.9
**Waist-to-hip ratio**	0.86±0.09	0.86±0.09	0.86±0.09	0.2
**Rs1916345**				
**N (M/W)**	10205(5351/4854)	4107(2124/1983)	451(269/182)	
**Age (years)**	54.4±9.9	54.8±10.0	54.6±9.8	
**BMI (kg/m^2^)**	27.5±4.8	27.6±4.9	27.8±5	0.6
**Weight (kg)**	81.1±16.3	80.7±16	82.6±16.5	0.7
**Waist (cm)**	92.5±14.2	92.5±14.3	94.3±14.2	0.5
**Waist-to-hip ratio**	0.86±0.09	0.86±0.09	0.88±0.09	0.8
**Rs2948694**				
**N (M/W)**	11844(6242/5602)	2722(1407/1315)	189(93/96)	
**Age (years)**	54.6±9.9	54.2±10	54.7±10.2	
**BMI (kg/m^2^)**	27.6±4.9	27.5±4.8	28.0±4.8	0.6
**Weight (kg)**	81.1±16.2	81±16.4	81.9±16.4	0.5
**Waist (cm)**	92.6±14.3	92.5±14.4	93.3±14.8	0.4
**Waist-to-hip ratio**	0.86±0.09	0.86±0.09	0.85±0.08	0.4
**Rs572169**				
**N (M/W)**	6535(3412/3123)	6444(3406/3038)	1630(848/782)	
**Age (years)**	54.6±9.9	54.5±10	54.6±9.6	
**BMI (kg/m^2^)**	27.6±4.9	27.6±4.9	27.6±4.8	0.9
**Weight (kg)**	80.9±16.3	81.3±16.2	81.2±16.5	0.3
**Waist (cm)**	92.6±14.3	92.8±14.3	92.4±14.2	0.7
**Waist-to-hip ratio**	0.86±0.09	0.86±0.09	0.86±0.09	0.1
**Rs495225**				
**N (M/W)**	7791(4109/3682)	5845(3045/2800)	1174(621/553)	
**Age (years)**	54.4±9.9	54.5±10	54.5±9.8	
**BMI (kg/m^2^)**	27.6±4.8	27.5±4.9	27.8±4.8	0.3
**Weight (kg)**	81.2±16.3	80.8±16.2	81.8±16.4	0.9
**Waist (cm)**	92.5±14.2	92.4±14.3	93.3±14.6	0.2
**Waist-to-hip ratio**	0.86±0.09	0.86±0.09	0.86±0.09	0.8
**Rs2922126**				
**N (M/W)**	6587(3485/3102)	6431(3374/3057)	1626(825/801)	
**Age (years)**	54.6±9.8	54.3±10	54.5±9.9	
**BMI (kg/m^2^)**	27.6±4.8	27.6±4.9	27.4±4.8	0.6
**Weight (kg)**	81±16.2	81.2±16.3	80.5±16.2	0.9
**Waist (cm)**	92.5±14.2	92.7±14.4	92.1±14.3	0.7
**Waist-to-hip ratio**	0.86±0.09	0.86±0.09	0.86±0.09	0.4

Data represents means ± SD. P-values were calculated using general linear model with age and sex as covariates. Subjects already diagnosed with type 2 diabetes at time of examination were excluded from the analyses. P_ADD_, *p*-values for additive analyses adjusted for sex and age. *Dominant model.

Four haplotypes were constructed from six of the variations located within the same LD block (Figure S1). A trend on global association was seen for waist-to-hip ratio (*p* = 0.06), without evidence for any specific haplotype causing the association (Table 2).

**Table 2:** Haplotype analysis of the identified *GHSR* haplotypes among 15,854 Danes on quantitative traits related to obesity.

Haplotype number	rs1403637	rs1916345	Rs509035	Rs2948694	Rs572169	Rs495225	Frequency (%)	*P-*value: Case-control BMI <25 kg/m^2^ vs. BMI >30 kg/m^2^	*P-*value: QT BMI	*P-*value: QT Waist	*P-*value: QT Waist-to-hip ratio	*P-*value: QT Weight
2	G	C	C	A	G	T	38	0.3	0.3	0.5	0.1	0.5
4	A	C	C	G	G	C	10	0.7	0.6	0.4	0.1	0.4
5	A	C	T	A	A	T	33	0.6	0.5	0.8	0.1	0.2
6	A	T	C	A	G	C	17	0.6	0.5	0.6	0.5	0.6
Global *p*-value:								0.8	0.4	0.5	0.06	0.2

The global *p*-value is an estimate for the overall effect of haplotypes in the statistical model and the specific *p*-value is an estimate of the effect of a specific haplotype compared to the effect of the remaining haplotypes combined. QT: Quantitative trait analysis.

### Mutation screening for rare *GHSR* variants

To discover potential rare variants we performed a mutational analysis of the coding region and the minimal promoter of *GHSR.* We identified six variants, of which five were known synonymous variants: Asp20Asp (rs2232165, minor allele frequency (MAF): 2.1%), Glu57Glu (rs495225, MAF: 24.1%), Leu149Leu (rs2232169, MAF: 2.6%), Arg159Arg (rs572169, MAF: 34.3%), Pro177Pro (rs4988509, MAF: 0.7%). These synonymous variants were not further investigated. The remaining variant was a rare mutation located in the promoter region (−151 C/T) and the mutation was identified in one obese individual.

### Family studies of the −151 C/T promoter mutation

**Figure 1:**
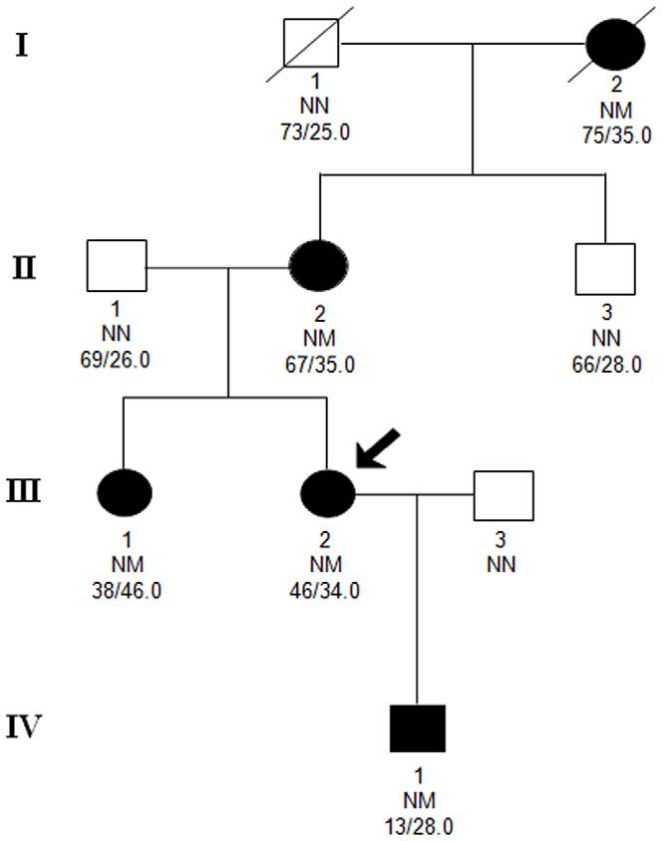
Pedigree of Danish family carrying the −151 *GHSR* promoter variant. Filled symbols indicate obese subjects. For subjects aged 18 years or older, obesity was defined as a BMI ≥30 kg/m^2^, for children international cut off points for obesity were used to define obesity (Cole, Bellizzi, et al. 2000 428/id). The index patient is indicated by the arrow. The roman number refers to generation and the text below each individual represents the following: family-specific identification number; genotype NN, no mutation; NM, heterozygous *GHSR* −151C>T mutation and age (years)/maximal BMI (kg/m^2^). DK-III-3 was not available for medical exams or interviews for this study. DK-X-Y (Danish family, generation X and person ID Y). The index patient (DK-III-2).

This -151 C/T promoter variant co-segregated with age-adjusted overweight or obesity in a Danish pedigree (Figure 1). The index patient, her son, and sister had all been overweight or obese since the onset of puberty and her mother since her early twenties. The index patient had type 2 diabetes and her sister had impaired glucose tolerance.

**Figure 2:**
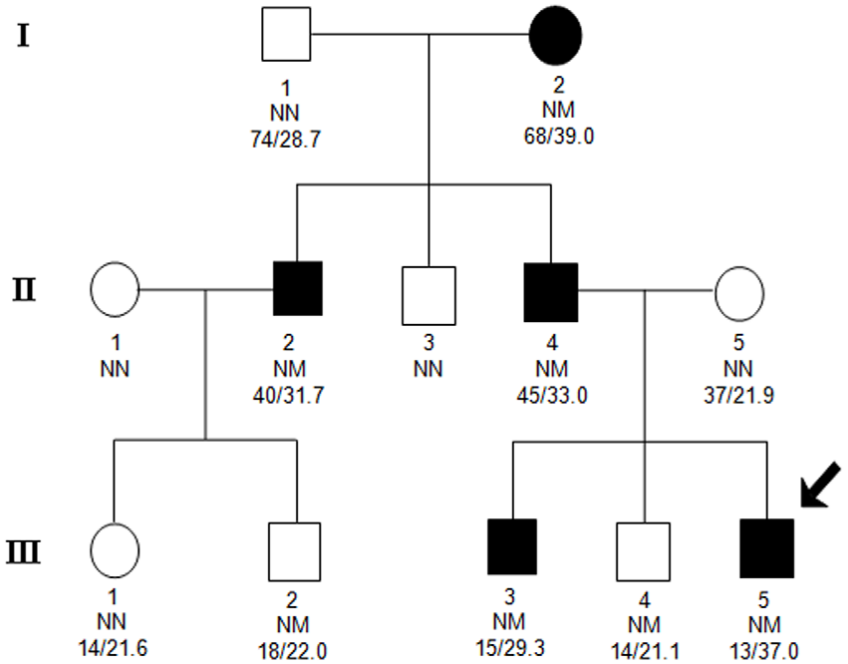
Pedigree of Czech family carrying the -151 *GHSR* promoter variant. Filled symbols indicate obese subjects. For subjects above 18 years obesity was defined as a BMI ≥30 kg/m^2^, obesity in children was defined as a BMI above 97th percentile for sex and age according to the Czech national references (27). The index patient is indicated by the arrow. The roman number refers to the generation and the text below each individual represents the following: family-specific identification number; genotype NN, no mutation; NM, heterozygous *GHSR* -151C>T mutation and age (years)/maximal BMI (kg/m^2^). All persons are represented with their maximal BMI and age at maximal BMI. CZ-X-Y (Czech family, generation X and person ID Y). CZ-II-3 has not been available for a medical exam or interview, but is reported by family members to be lean.

In 289 obese Czech children, we identified an additional heterozygous carrier of the -151C/T mutation and co-segregation was seen for all obese individuals in the Czech pedigree (Figure 2). The children CZ-III-2 and CZ-III-4 (Figure 2) were also carriers of the mutation with BMIs of 22.0 (Z-score: +0.04) and 21.1 (Z-score: +0.3) kg/m^2^, respectively, which is above the age and sex-adjusted 50^th^ percentile. When linkage analysis was performed in the Danish and Czech pedigrees they showed a LOD score for obesity at the *GHSR* locus of Z  = 1.7.

### *In vitro* analysis of the *GHRS* -151C>T mutation

**Figure 3:**
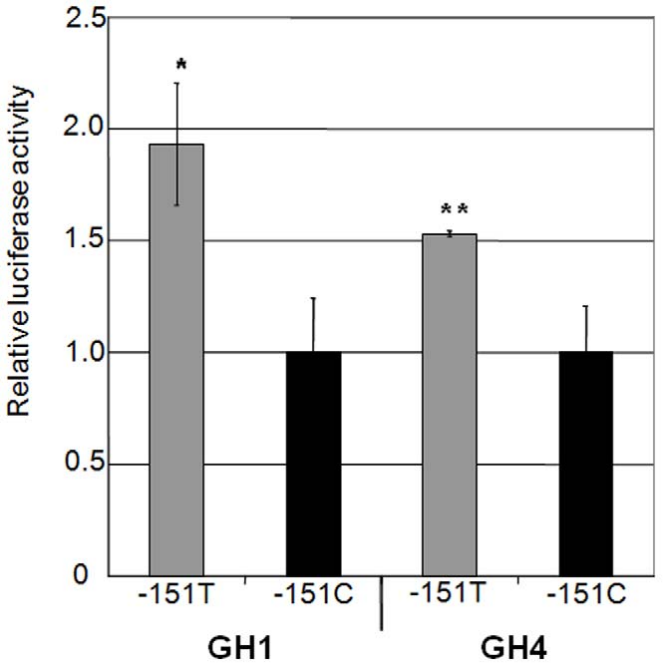
Transcriptional activity of the −151 wild type (C) and the variant (T) *GHSR* promoter. Activity of *GHSR* –151T versus −151C −643/pGL3 GHSR gene constructs in GH1 and GH4 cells. Values represent the mean ± SD of at least 3 measurements of luciferase activity normalised with CMV-renilla activity expressed as fold induction relative to the activity of the wild type promoter. P values: * *p* = 0.0005 and ** *p* = 0.02, calculated by t-test for independent samples.

**Figure 4:**
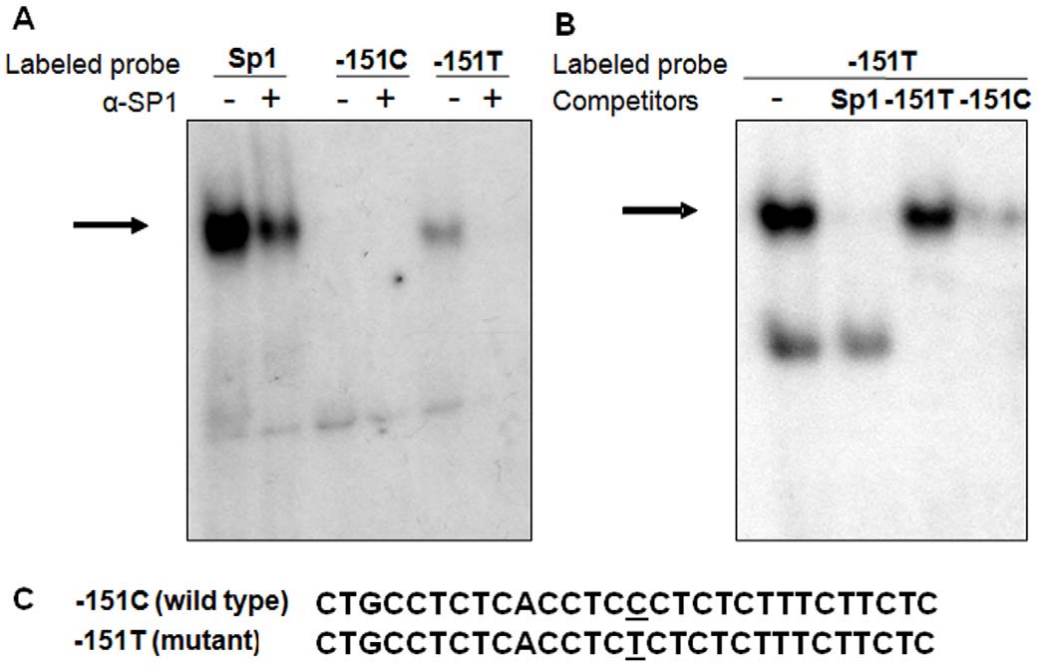
Effect of the *GHSR* -151T variant on the affinity of the Sp1-binding site by EMSAs. GH4 nuclear extracts incubated with labeled oligonucleotide probes containing an SP1 consensus site, the wild type GHSR -151C sequence, or the mutated GHSR –151T sequence. A: anti SP1 antibody (α-SP1) was added to the reaction mix. B: binding to the –151T probe was competed with 200-fold molar excess of indicated unlabeled competitor probes. An arrow points to the complex exhibiting sequence-selective binding to the –151T probe. **C**: Sequences of wild type and mutant oligonucleotide probes.

Studies of the transcriptional activity of the -151T promoter compared to a wild type showed an increased transcriptional activity measured as renilla-controlled luciferase activity in constructs expressing the 643 nucleotides upstream of the translation initiation site (Figure 3). *In silico* analyses predicted a change in the binding of several transcription factors including the introduction of a Sp1-like binding site caused by the introduction of the -151 variant. Electro-mobility shift assays (EMSAs) showed that the -151T mutant sequence created a high affinity binding site for a nuclear complex displaced by anti-Sp1 antibody and by competition with an Sp1 consensus oligonucleotides (Figure 4). Displacement of the labelled mutant probe was not observed with an unrelated antiserum or unlabeled Egr1 and Ets1 probes (data not shown).

### The *GHRS* -151C>T mutation and relationships to anthropometrics, biochemical variables and health behavior

Due to the established functionality of the -151T *GHSR* mutation the physiological and biochemical profile of carriers of the -151T transcription-activating mutation was examined in the Danish and Czech families. Using the TFEQ we did not see consistent measures of hunger, restrain or disinhibition for carriers of the -151T mutation (Table S2). Also, birth weight and height as well as psychosocial and intellectual development were assessed as being normal by self-report of mutation carriers. None of the selected biochemical measures for family members were abnormal (Table S3). Neither the meal test revealed an abnormal response (data not shown).

### Search for potential impact of the -151 promoter mutation at the population level

Despite the lack of consistent linkage with obesity of the -151 promoter mutation in the investigated families, we examined the possible association of the variant with obesity in the general population. Due to the low allele frequency only 26 heterozygous and no homozygous individuals were identified (Table 3). Applying a dominant model of inheritance we found no association between mutation carriers and prevalence of obesity (Table 3) or quantitative measures of body fat (Table 1).

**Table 3:** Obesity case-control study of the −151 C/T *GHSR* promoter variant in Danes from the Inter99 study, the Danish ADDITION Screening Study and the Steno Diabetes Center.

	BMI <25 kg/m^2^	BMI 25 ≥<30 kg/m^2^	BMI ≥30 kg/m^2^	P_Fishers (dom)_	OR (CI)	P_GLM_ _(dom)_
CC	2855 (99.9)	6086 (99.9)	3852 (99.9)			
CT	4 (0.1)	14 (0.1)	5 (0.1)			
TT	0	0	0			
MAF	0.1 (0.0–0.1)	0.1 (0.1–0.2)	0.1 (0.0–0.1)	1.0	0.93 (0.2–4.67)	-
GC				-	0.61 (0.14–2.58)	0.5

Data are number of subjects with each genotype (% of each group). P_Fishers_: Fisher's exact test comparing allele frequencies and genotype distribution between lean (BMI <25 kg/m^2^) and obese (BMI ≥30 kg/m^2^) subjects. P_GLM_: General linear model (GLM) adjusted for sex and age comparing differences in genotype distribution Genotype distribution (GD). Minor allele frequency (MAF). OR: is the increased risk pr. allele of being obese. Only analyses assuming a dominant model (dom) have been performed due to the low allele frequency. BMI, body mass index.

When evaluating these results it should, however, be kept in mind that no correction for multiple testing was made.

## Discussion

Haplotypes composed of common variants within the *GHSR* locus showed borderline association with waist-to-hip ratio, despite the lack of association between the same phenotypes and the individual variants. *GHSR* haplotypes have previously been associated with obesity [8], yet the only overlapping variant in these haplotype studies was rs572169. This variant has previously been found to associate with obesity on its own [8], [9]. However, in line with our findings, this could not be replicated in a German study sample. Thus, common *GHSR* variants are most likely not major contributors to obesity among Caucasian individuals.

Previous studies have implied not only an effect of common *GHSR* variants on the pathogenesis of obesity but also of rare variants [10]. From the mutation screening the mostly likely functional variant identified was the -151C/T mutation located in the *GHSR* promoter. It was further investigated in two families (a Danish and Czech). Whilst we found complete co-segregation with obesity or overweight within the Danish family, only incomplete co-segregation was demonstrated within the Czech family possibly due to incomplete penetrance of the variant. The mutation increased the transcriptional activity of *GHSR*. This increase is probably due to an introduction of a highly specific SP-1-like site as we have shown that other candidate consensus sequences, such as Egr1 and Ets1, predicted to bind selectively to the -151T mutant promoter by our *in silico* analyses did not bind.

The increased *GHSR* transcriptional activity and thereby the increased expression of GHSR would be expected to lead to increased signaling independent of the ghrelin hormone due to the high constitutive activity of this receptor, resulting in a possible increase in appetite and decrease in energy expenditure. Therefore, changes in GHSR expression could play a major role in appetite regulation and energy expenditure as seen in the Danish pedigree. Yet, this hypothesis was not supported by the outcome of TFEQ.

Despite the functionality of the -151C/T *GHSR* mutation we did not find a significant impact of this mutation in the general population of adult Danes. This could be due to the low number of carriers. Whilst, it can not be ruled out that this rare *GHSR* promoter variant may contribute modestly to the pathogenesis of obesity it would require a much larger study sample to investigate this.

In summary, common variants in *GHSR* did not associate with measures of obesity or overweight in the general population of Danes. However, a rare promoter variant, which exerts a significant functional effect on the ghrelin receptor transcription, showed partial co-segregation with obesity and overweight when examined in 2 pedigrees of whites.

The study is part of the DanORC project (http://www.danorc.dk) and was additionally supported by the University of Copenhagen, the Danish Diabetes Association, The European Union (HEPADIP, grant no. LSHM-CT-2005-018734), the Danish Medical Research Council, the Danish Agency for Science Technology and Innovation (grant no. 271-06-0539), the Czech research project of MSM No. 0021620814 and an European Society of Paediatric Endocrinology (ESPE) research fellowship sponsored by Novo Nordisk.

## Supporting Information

Figure S1 LD structure of common variants genotyped in the *GHSR* locus. A; D' value. B; R^2^ value. Black bar is marking the LD-block located in the region tagged by variants.
(5.95 MB TIF)

Table S1 Association of variants in *GHSR* among Danish study participants recruited from the Inter99 study, the Danish ADDITION Screening Study and the Steno Diabetes Center. Data are number of subjects with each genotype (% of each group). P_Fishers_: Fisher's exact test comparing allele frequencies and genotype distribution between lean (BMI <25 kg/m^2^) and obese (BMI ≥30 kg/m^2^) subjects. P_GLM_: General linear model (GLM) adjusted for sex and age comparing differences in genotype distribution Genotype distribution (GD). Minor allele frequency (MAF). OR: is the increased risk pr. allele of being obese.
(0.10 MB DOC)

Table S2 Three Factor Eating Questionnaire (TFEQ) values from the Danish and Czech family members. DK-X-Y (Danish family, generation X and person ID Y). CZ-X-Y (Czech family, generation X and person ID Y).W, woman, M, man, nd =  not determined. Maximum scores: Restraint  = 21, Disinhibition  = 16, and Hunger  = 14 (30).
(0.06 MB DOC)

Table S3 Biochemical variables from Danish and Czech families. Luteinizing hormone (LH), follicle stimulating hormone (FSH). DK-X-Y (Danish family, generation X and person ID Y). CZ-X-Y (Czech family, generation X and person ID Y).
(0.05 MB DOC)
