# Supplementary figures and images for: Family and Population-Based Studies of Variation within the Ghrelin Receptor Locus in Relation to Measures of Obesity
Source: PLoS One. 2010 Apr 9;5(4):e10084. doi: 10.1371/journal.pone.0010084 (PMC2852411; doi:10.1371/journal.pone.0010084)

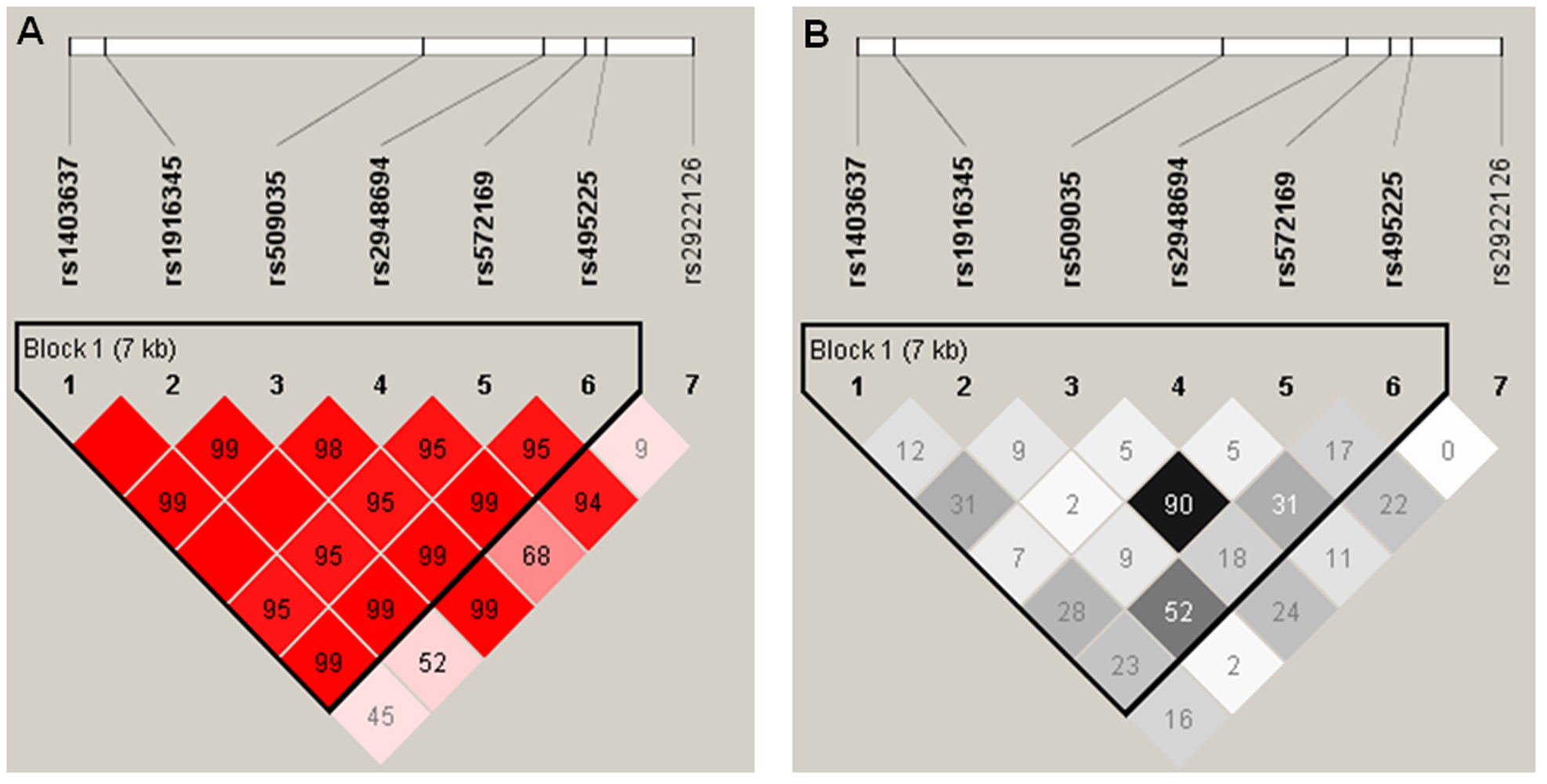

Supplement: Figure S1 — LD structure of common variants genotyped in the GHSR locus. A; D' value. B; R2 value. Black bar is marking the LD-block located in the region tagged by variants. (5.95 MB TIF) [file pone.0010084.s001.tif]
