# Supplementary material for: Family and Population-Based Studies of Variation within the Ghrelin Receptor Locus in Relation to Measures of Obesity
Source: PLoS One. 2010 Apr 9;5(4):e10084. doi: 10.1371/journal.pone.0010084 (PMC2852411; doi:10.1371/journal.pone.0010084)
Supplement: Table S1 — Association of variants in GHSR among Danish study participants recruited from the Inter99 study, the Danish ADDITION Screening Study and the Steno Diabetes Center. Data are number of subjects with each genotype (% of each group). PFishers: Fisher's exact test comparing allele frequencies and genotype distribution between lean (BMI <25 kg/m2) and obese (BMI ≥30 kg/m2) subjects. PGLM: General linear model (GLM) adjusted for sex and age comparing differences in genotype distribution Genotype distribution (GD). Minor allele frequency (MAF). OR: is the increased risk pr. allele of being obese. (0.10 MB DOC) [file pone.0010084.s002.doc]

|  | BMI < 25 kg/m^2^ | BMI ≥ 30 kg/m^2^ | P_Fishers_ | OR (CI) | P_GLM_ |
| --- | --- | --- | --- | --- | --- |
| **Rs1403637** |  |  |  |  |  |
| **AA** | 1130 (37.9) | 1567 (39.1) |  |  |  |
| **AG** | 1391 (46.6) | 1859 (46.4) |  |  |  |
| **GG** | 462 (15.5) | 581 (14.5) |  |  |  |
| **MAF** | 38.8 (37.6-40.0) | 37.7 (36.6-38.8) | 0.19 | 0.95 (0.89-1.02) |  |
| **GD** |  |  | 0.41 | 0.95 (0.88-1.03) | 0.24 |
| **Rs1916345** |  |  |  |  |  |
| **CC** | 2072 (70.3) | 2718 (68.8) |  |  |  |
| **CT** | 794 (26.9) | 1109 (28.1) |  |  |  |
| **TT** | 83 (2.8) | 126 (3.2) |  |  |  |
| **MAF** | 16.3 (15.3-17.2) | 17.2 (16.4-18.0) | 0.15 | 1.07 (0.98-1.17) |  |
| **GD** |  |  | 0.35 | 1.05 (0.95-1.16) | 0.36 |
| **Rs2948694** |  |  |  |  |  |
| **AA** | 2361 (80.1) | 3196 (80.1) |  |  |  |
| **AG** | 548 (18.6) | 737 (18.5) |  |  |  |
| **GG** | 39 (1.3) | 59 (1.5) |  |  |  |
| **MAF** | 10.6 (9.8-11.4) | 10.7 (10.0-11.4) | 0.87 | 1.01 (0.9-1.13) |  |
| **GD** |  |  | 0.87 | 1.04 (0.92-1.18) | 0.5 |
| **Rs572169** |  |  |  |  |  |
| **GG** | 1287 (44.9) | 1784 (45) |  |  |  |
| **GA** | 1268 (44.2) | 1732 (43.7) |  |  |  |
| **AA** | 311 (10.9) | 450 (11.3) |  |  |  |
| **MAF** | 33.0 (31.8-34.2) | 33.2 (32.1-34.2) | 0.81 | 1.01 (0.94-1.09) |  |
| **GD** |  |  | 0.78 | 1.01 (0.93-1.09) | 0.89 |
| **Rs2922126** |  |  |  |  |  |
| **TT** | 1285 (43.6) | 1789 (45.4) |  |  |  |
| **TA** | 1318 (44.7) | 1734 (44) |  |  |  |
| **AA** | 343 (11.6) | 416 (10.6) |  |  |  |
| **MAF** | 34.0 (32.8-35.2) | 32.6 (31.5-33.6) | 0.08 | 0.94 (0.87-1.01) |  |
| **GD** |  |  | 0.2 | 0.93 (0.86-1.01) | 0.1 |
| **Rs495225** |  |  |  |  |  |
| **TT** | 1586 (53.3) | 2089 (52.4) |  |  |  |
| **TC** | 1157 (38.9) | 1553 (39) |  |  |  |
| **CC** | 230 (7.7) | 342 (8.6) |  |  |  |
| **MAF** | 27.2 (26.1-28.3) | 28.1 (27.1-29.1) | 0.26 | 1.05 (0.97 - 1.13) |  |
| **GD** |  |  | 0.42 | 1.05 (0.96-1.14) | 0.29 |
