# Supplementary material for: Family and Population-Based Studies of Variation within the Ghrelin Receptor Locus in Relation to Measures of Obesity
Source: PLoS One. 2010 Apr 9;5(4):e10084. doi: 10.1371/journal.pone.0010084 (PMC2852411; doi:10.1371/journal.pone.0010084)
Supplement: Table S2 — Three Factor Eating Questionnaire (TFEQ) values from the Danish and Czech family members. DK-X-Y (Danish family, generation X and person ID Y). CZ-X-Y (Czech family, generation X and person ID Y).W, woman, M, man, nd = not determined. Maximum scores: Restraint = 21, Disinhibition = 16, and Hunger = 14 (30). (0.06 MB DOC) [file pone.0010084.s003.doc]

| **ID** | **Sex** | **Age** | **BMI** | **Restraint** | **Disinhibition** | **Hunger** |
| --- | --- | --- | --- | --- | --- | --- |
| DK-II-2 | W | 69 | 30.7 | 12 | 7 | 6 |
| DK-III-1 | W | 38 | 45.9 | nd | nd | nd |
| DK-III-2 | W | 48 | 34.2 | 9 | 10 | 14 |
| DK-IV-1 | M | 19 | 23.4 | 14 | 6 | 2 |
| CZ-I-1 | W | 68 | 28.7 | nd | nd | nd |
| CZ-I-2 | M | 74 | 35.4 | 6 | 2 | 0 |
| CZ-II-1 | W | 38 | 24.7 | 5 | 5 | 1 |
| CZ-II-2 | M | 40 | 31.7 | 2 | 7 | 4 |
| CZ-II-3 | M | nd | nd | nd | nd | nd |
| CZ-II-4 | M | 45 | 33.0 | 4 | 3 | 0 |
| CZ-II-5 | W | 37 | 21.9 | 17 | 2 | 2 |
| CZ-III-1 | W | 14 | 21.6 | 2 | 1 | 0 |
| CZ-III-2 | M | 18 | 22.0 | 1 | 3 | 2 |
| CZ-III-3 | M | 18 | 23.7 | 6 | 14 | 11 |
| CZ-III-4 | M | 15 | 21.1 | 11 | 3 | 2 |
| CZ-III-5 | M | 13 | 37.0 | 4 | 7 | 10 |
