# Supplementary material for: Family and Population-Based Studies of Variation within the Ghrelin Receptor Locus in Relation to Measures of Obesity
Source: PLoS One. 2010 Apr 9;5(4):e10084. doi: 10.1371/journal.pone.0010084 (PMC2852411; doi:10.1371/journal.pone.0010084)
Supplement: Table S3 — Biochemical variables from Danish and Czech families. Luteinizing hormone (LH), follicle stimulating hormone (FSH). DK-X-Y (Danish family, generation X and person ID Y). CZ-X-Y (Czech family, generation X and person ID Y). (0.05 MB DOC) [file pone.0010084.s004.doc]

|  | DK-II-2 | DK-III-2 | DK-IV-1 | CZ-III-5 | CZ-II-5 | CZ-III-4 |
| --- | --- | --- | --- | --- | --- | --- |
| Mutation (yes/no) | Yes | Yes | Yes | Yes | No | No |
| Age (year) | 69 | 48 | 19 | 13 | 37 | 15 |
| Sex | F | F | M | M | F | M |
| BMI kg/m^2^ | 30.7 | 34.2 | 23.4 | 37 | 21.9 | 21.1 |
| Triacylglycerol (mmol/L) | 1.0 | 1.4 | 0.8 | 2.6 | 0.8 | 1.3 |
| Fasting p-glucose mM | 5.4 | 8.1 (T2D) | 5.2 | 4.4 | 3.6 | 4.4 |
| LH (U/L) | 20.8 | 20.7 | 2.2 | 3.2 | 17.3 | 3.8 |
| FSH (U/L) | 59.4 | 28.6 | 2.3 | 3.7 | 6.2 | 3.5 |
| IGF-BP3 (ng/ml) | 2614 | 4032 | 4365 | 6.2 | 4.5 | 5.5 |
| Leptin (ng/mL) | 39 | 50 | 3 | nd | nd | nd |
